# Neurogenic Pulmonary Edema Following a Seizure: A Case Report and Literature Review

**DOI:** 10.1155/2019/6867042

**Published:** 2019-10-09

**Authors:** Mostafa Suhail Najim, Riyadh Ali Mohammed Hammamy, Sreethish Sasi

**Affiliations:** Department of Internal Medicine, Hamad Medical Corporation, Doha, Qatar

## Abstract

Acute pulmonary edema is one of the frequent causes of dyspnea encountered in everyday practice. It is broadly attributed to be either cardiogenic or noncardiogenic. It is usually treated with diuretics in addition to other medications depending on the underlying pathology. Here, we report a case of a female patient who presented with shortness of breath after developing a seizure. Further investigations excluded cardiogenic etiology and showed critically low phenytoin level. It improved within 48 h of supportive care without giving diuretics favoring the diagnosis of neurogenic pulmonary edema as the primary pathology. The goal of our case report is to keep neurogenic pulmonary edema in mind, and hence provide the appropriate management, when dealing with similar cases.

## 1. Introduction

Neurogenic pulmonary edema (NPE) refers to acute pulmonary edema developing within hours after an acute injury to the central nervous system [1]. NPE is usually overlooked due to its low prevalence, and absence of clear diagnostic criteria [2]. It occurs following a wide spectrum of neurological insults with cerebral hemorrhage being the commonest and only 2% are reported following a convulsion [3]. Despite this rarity, almost 80% of epileptic patients who died unexpectedly after a seizure were noted to have NPE [4]. Clinically, NPE is broadly classified into early (within minutes to hours) and delayed (12–24 h) following a neurologic insult. It presents with dyspnea, hypoxia, pink frothy sputum, and bilateral crackles on chest auscultation that correlates with bilateral chest radiograph infiltrates [2]. It is usually diagnosed after the exclusion of clear cardiogenic and intrinsic pulmonary factors contributing to the development of acute pulmonary edema [1].

## 2. Case Report

A 51-year-old lady presented with cough and shortness of breath. The symptoms started in the morning after waking up from sleeping. The cough was productive of pink-colored sputum and she was using accessory muscles to breathe. The family members noticed that she was also drowsy, confused, and not responding to them for about 30 min. Afterwards, she was fully aware of the surroundings; however, she could not recall what happened. They measured her blood glucose and it was 8.5 mmol/L. The past medical history was significant for a resected right temporal meningioma 3 years back and type 2 diabetes mellitus with well controlled HbA1c. Home medications are phenytoin 200 mg twice daily, lamotrigine 200 mg three times daily, escitalopram 10 mg once daily, and metformin/sitagliptin 1000/50 mg twice daily. The patient is known to experience seizures despite those anti-epileptic medications.

On examination, she was afebrile (37.3°C), hypotensive (89/47 mmHg), and hypoxic (82% oxygen saturation on room air). Respiratory exam showed bilateral crackles up to the mid zones bilaterally. Neurological examination did not reveal any abnormality. The initial laboratory tests ordered upon admission are shown in Table 1.

### 2.1. Blood Gases

*Arterial Blood Gases (ABG) on Room Air*. pH 7.41 (7.35–7.45), PaO_2_ 48.8 mmHg (83–108), PaCO_2_ 35 mmHg (35–45), HCO_3_ 22.5 mmol/L (21–28), PaO_2_/FiO_2_ ratio: 232 (normal ratio is approximately 500).

*ABG on Room Air after 2 Days*. pH 7.43 (7.35–7.45), PaO_2_ 90 mmHg (83–108), PaCO_2_ 36 mmHg (35–45), HCO_3_ 24.5 mmol/L (21–28), PaO_2_/FiO_2_ ratio: 429 (normal ratio is approximately 500).

### 2.2. Diagnostic Work-Up

**Figure 1:**
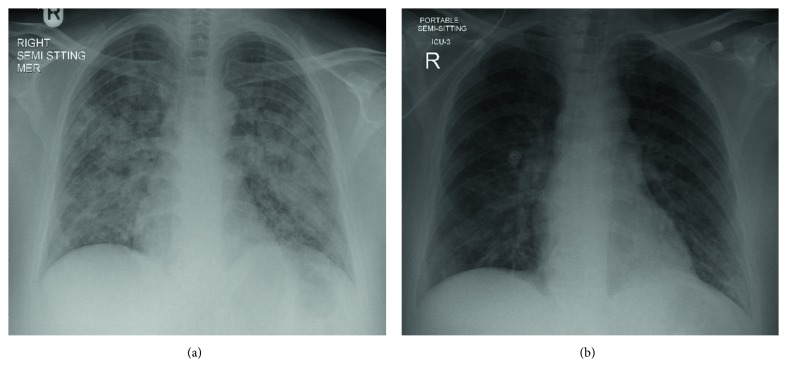
(a) CXR on admission showing multiple confluent and patchy air space opacities noticed diffusely involving both lung fields. (b) CXR after 2 days showing the bilateral lung opacities appear significantly resolved as compared to the previous chest radiograph.

*CXR on Admission*. Multiple confluent and patchy air space opacities noticed diffusely involving both lung fields (Figure 1).

*CXR after 2 Days*. Bilateral lung opacities appear significantly resolved as compared to the previous chest radiograph (Figure 1).

**Figure 2:**
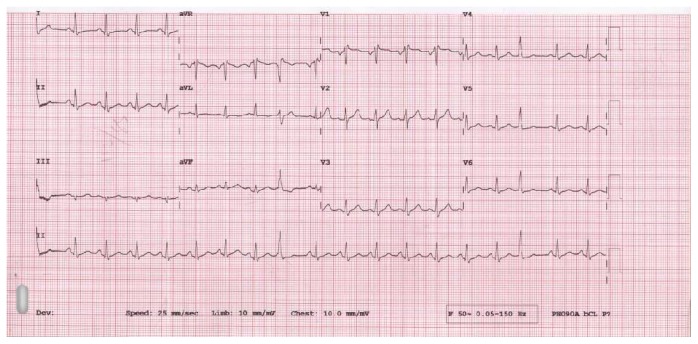
ECG showing sinus tachycardia with premature ventricular complexes.

*ECG*. Sinus tachycardia with premature ventricular complexes (Figure 2).

*Trans-Thoracic Echocardiography*. Good global contractility and no resting regional wall motion abnormality. Normal Left ventricle systolic function. Ejection fraction 58%–60%.

### 2.3. Hospital Course

The patient received 1.5 L of intravenous normal saline boluses, she was connected to noninvasive ventilator—Continuous positive airway pressure (CPAP) machine with empirical IV. antibiotic to cover aspiration pneumonia and admitted to MICU for observation. There, she stayed for 2 days and showed marked improvement clinically and in the requirements of oxygen. Repeated CXR showed dramatic resolution of bilateral lung opacities without giving furosemide (Figure 1). Compliance to anti-convulsant medications was emphasized, and she was maintaining oxygen saturation above 95% on room air upon discharge.

## 3. Discussion

The pathophysiology behind NPE is not fully understood. Increased intracranial pressure (ICP) is thought to be involved in the pathogenesis of NPE via catecholamine release that causes intense pulmonary and systemic vasoconstriction leading to an increase in capillary hydrostatic pressure and capillary permeability [5–7]. This theory is supported by the demonstration of NPE mitigation when using sympatholytic agents in animal studies [8]. Furthermore, a previous case report had demonstrated a noncardiogenic pulmonary edema as the sole clinical presentation of pheochromocytoma and it was attributed to the catecholamines surge [9]. Another proposed mechanism of NPE involves the central release of the inflammatory mediators secondary to endothelial damage into the systemic circulation. This increases the pulmonary capillary permeability leading to NPE formation [1]. Hypoxemia might be a contributing factor for the development of NPE. Severe ictal hypoxemia has been reported in different types of seizures especially the generalized convulsive ones [10]. Hypoxemia causes peripheral and pulmonary vasoconstriction with shunting of blood to pulmonary vasculature further compromising pulmonary hemodynamics. Hypoxemia also interferes with the alveolar epithelial Na, K-ATPase active transport that is necessary for clearance of NPE fluid [11].

In one retrospective study that involves reviewing chest CT scan for patients presenting with seizures as part of their emergency diagnostics, it was found that NPE was more frequent in cases of generalized convulsive seizures [12]. Recurrent NPE was also reported as the cause of two consecutive intensive care unit admissions following generalized convulsive seizures [13].

NPE is a diagnosis of exclusion. Cardiac etiology should be ruled out first. In addition, it requires the demonstration of hypoxemia and bilateral pulmonary infiltrates, in the presence of a genuine central nervous system insult raising the intracranial pressure [2].

In our case report, there are several clues to diagnose NPE. The witnessed postictal phase, the shortness of breath with oxygen desaturation, the bilateral infiltrates on chest radiograph, the rapid resolution within 48 h without diuretics, the critically subtherapeutic serum level of phenytoin, and the exclusion of cardiac causes, all have made the diagnosis of NPE on the top of the differential diagnosis list.

Aspiration pneumonia is one of the most relevant diagnosis that needs to be excluded before confirming the diagnosis of NPE [1]. The clinical, laboratory, and imaging findings that help to differentiate between the two are summarized in Table 2. In the setting of extubation, negative pressure pulmonary edema may mimic NPE[14].

## 4. Conclusion

Acute pulmonary edema is most commonly cardiogenic in origin. However, noncardiogenic causes should always be investigated. Diuretics, in addition to specific disease-related medications, are implemented in the management plan except in selected cases as in neurogenic pulmonary edema where supportive care is the mainstay of treatment.

## Figures and Tables

**Table 1:** Laboratory tests^*∗*^.

Laboratory test	Patient's values	Normal reference range
*General hematology*		
White blood cells (10^3^/*µ*L)	9.6 *Normal*	4–10
Hemoglobin (g/dL)	10.9	12–15
Platelet (10^3^/*µ*L)	340	150–400

*General chemistry*		
Urea (mmol/L)	5.7	2.76–8.07
Creatinine (*µ*mol/L)	31	53–97
Sodium (mmol/L)	138	135–145
Potassium (mmol/L)	4.2	3.6–5.1
Chloride (mmol/L)	100	96–110
Magnesium (mmol/L)	0.77	0.66–1.07
Glucose (mmol/L)	11.2	3.3–5.5
Bicarbonate (mmol/L)	22.9	24–30
Albumin (g/L)	40	35–50
Corrected calcium (mmol/L)	2.16	2.1–2.6
Phosphorus (mmol/L)	1.08	0.87–1.45
Bilirubin total (*µ*mol/L)	4.5	3.5–24
ALT (U/L)	12	0–30
AST (U/L)	15	0–31
NT-pro BNP (pg/mL)	717	0–300
Troponin T highly sensitive (ng/L) three sets	24.8 → 20.6 → 16.4	0–14
C-reactive protein (mg/L)	23 *High*	0–5
Procalcitonin (ng/mL)	0.26 *Normal*	0–0.5
Lactic acid (mmol/L)	1.5	0.5–1.6

*Microbiology*		
Blood culture (2 sets)	*Negative*	—
Sputum culture	*Negative*	—

*Drug level*		
Phenytoin level (*µ*mol/L)	3.6 *Critical Low*	40–79

^*∗*^Lamotrigine and escitalopram levels are not available.

**Table 2:** Neurogenic pulmonary edema (NPE) vs. aspiration pneumonia (AP) [1].

Diagnosis	Onset	Fever	PaO_2_/FiO_2_ ratio	Chest X-ray findings	WBC	CRP	Procalcitonin	Evolution duration
NPE	Hours	Yes/No	↓	Bilateral	Normal/↑	Normal/↑	Normal	1–3 days
AP	24 h	Yes	Normal/↓	Uni/bilateral	↑	↑	Normal/↑	1–3 weeks

PaO_2_: arterial partial pressure of oxygen; FiO_2_: inspiratory fraction of oxygen; WBC: white blood cells; CRP: C-reactive protein; PCT: procalcitonin.
